# Supplementary material for: Role of Epidermal Growth Factor Receptor-Specific CAR-T Cells in the Suppression of Esophageal Squamous Cell Carcinoma
Source: Cancers (Basel). 2022 Dec 7;14(24):6021. doi: 10.3390/cancers14246021 (PMC9775531; doi:10.3390/cancers14246021)
Supplement: Supplementary file 1 [file cancers-14-06021-s001.zip › cancers-1955814-Supplementary.pdf]

cancers

MDPI

Supplementary Material: Role of Epidermal Growth Factor Receptor-Specific CAR-T Cells in the Suppression of Esophageal Squamous Cell Carcinoma

Chen Cheng, Heyang Cui, Huijuan Liu, Yueguang Wu, Ning Ding, Yongjia Weng, Weimin Zhang * and Yongping Cui,*

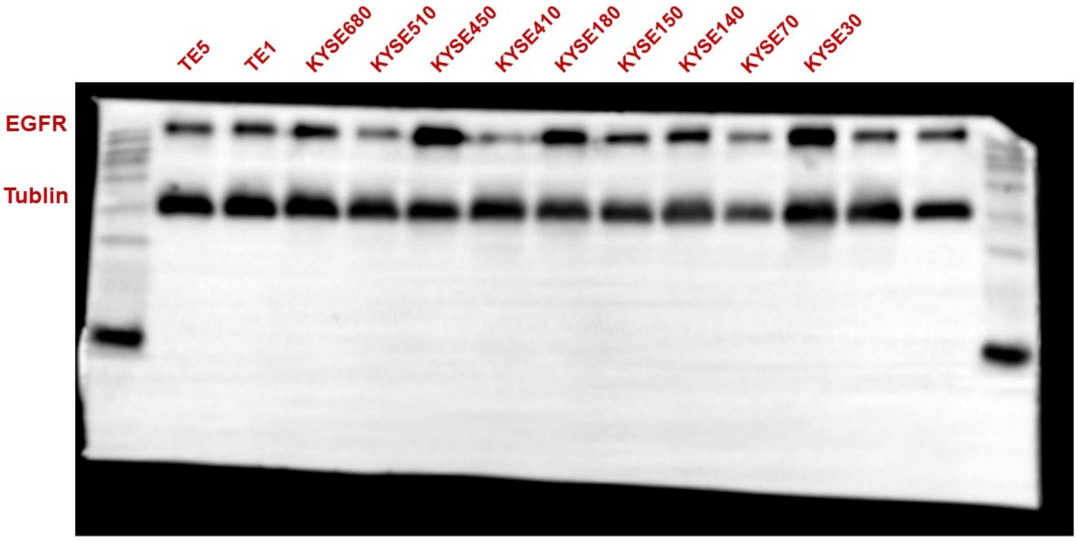

Figure S1. Uncropped Western blot image Figure 2C.
